# Cases of Hysteria from Indigestion

**Published:** 1818-07

**Authors:** 


*For the London Medical and Physical Journal.*

### *Cases of Hysteria from Indigestion;* by B., M. D.

THE following cases may, perhaps, appear interesting enough to be recorded in your Journal. They are selected from many others, and are drawn up as concisely as the intention of the communication would allow.

*November.*—Miss G., tall, melancholic temperament, great sensibility, and who has experienced much mental distress, complains of violent palpitation of the heart, from whence she fancies her illness arises, and which she has perceived some six months back. She suffers much from frequent head-ach, particularly from pain and weight over her eyes, a constant gnawing sensation in the stomach, with pain darting between the shoulders, and a numbness of the right arm; her nights are very restless, has constant sickness in the morning, oppression and distension of the stomach after meals, more especially in the evening, at which time, she is much tormented with heart-burn. Pulse is weak and hurried; tongue very foul; bowels costive; the evacuations dark-coloured and offensive; urine scanty; catamenia regular and abundant; appetite has entirely deserted her, and she is never free from slight febrile symptoms. She is, what is called, very nervous, any surprise immediately producing a fit of hysteria. On examination, the liver felt enlarged, she could not bear the slightest pressure on the right hypochondrium; and, when made a little forcibly, it immediately brought on the hysteric paroxysm,—the heart beating so as to be heard. Eight leeches were ordered to be applied to the right side, which was afterwards covered with a blister: she took a powder of calomel and jalap every other night, with some neutral salt the next morning; the common saline mixture in a state of effervescence, with gtt. x. sp. æther. sul. every six hours. This plan was pursued for a fortnight, the leeches and blister being twice repeated; by that time, the palpitation of the heart and dyspepsia were removed. A course of bitters and steel would have completed the cure, if she could have recovered her serenity of mind.

*January.*—Miss J., aged 16, pale, delicate, and of sedentary habits, complains of violent palpitations of the heart, which was first perceived in March last; it had been pronounced an organic disease, and, in consequence, she has taken for some time back the Tr. digitalis, which has neither retarded the palpitation nor appeased a cough, which is now very troublesome. On enquiry, I received the following account of herself. She feels excessively weak and overcome on the slightest exertion; has an occasional difficulty of breathing and sense of suffocation, a continual head-ach, especially across the forehead; pain in the stomach, darting between the shoulders and the right hypochondrium; constant sickness in a morning, with want of appetite for breakfast; a very unpleasant sense of fulness after meals; much feeling of weariness in an evening; but the moment she lies down, all desire for sleep goes off. Pulse is quick and irregular, but it does not synchronize with the motion of the heart, which is very rapid and powerful; tongue is very foul; the bowels costive; the stools the colour of pitch; the urine is laden with urea; catamenia more frequent than natural, and the discharge pale and scanty: her spirits are very good, but surprise brings on some strong symptoms of hysteria. There appeared a considerable congestion in the liver; a very acute pain was felt on pressure, "flying (to use her own terms) to the heart," and its motion was certainly very much accelerated. Eight leeches were applied to the right side, and a large blister after their operation; pills of ext. coloc. 60, with pulv. scammon. c̄ hyd. submur. were given every other night, and a saline opening draught the next morning; the common saline mixture three times a day. For some time, she was considerably better in every respect, but, venturing (I suppose) too early on a course of tonics, all her symptoms returned, and the hysteria and palpitation were more violent than ever.—Death was not unfrequently deemed close at hand by her attendants: recourse was again had to her former medicines, and, after more copious discharges by stool than I have ever known, she gradually amended. I have little doubt now of her health being perfectly re-established by country air and diet, and attention to the alvine discharge.

I find I shall trespass too much upon your pages, and will, therefore, close this communication. I will continue the cases, or not, as you may judge expedient.

*May*, 1818.

